# Epidemiology and genetic characteristics of coxsackievirus A16 associated with hand-foot-mouth disease in Hangzhou city, Zhejiang province from 2021 to 2024

**DOI:** 10.3389/fmicb.2025.1698485

**Published:** 2025-11-14

**Authors:** Qihao Xu, Xiaofeng Qiu, Yue Yu, Yanping Wen, Guozhong Zhang, Jun Li

**Affiliations:** 1 Hangzhou Center for Disease Control and Prevention (Hangzhou Health Supervision Institution), Hangzhou, China; 2 Zhejiang Key Laboratory of Multi-Omics in Infection and Immunity, Hangzhou, China

**Keywords:** coxsackievirus A16, B1c, molecular epidemiology, HFMD, Hangzhou

## Abstract

Hand-foot-mouth disease (HFMD) is a common acute infectious disease in children caused by various enteroviruses (EV). CV-A16 is one of the main pathogens of HFMD, leading to multiple outbreaks of HFMD worldwide, especially in the Asia-Pacific region. This study aims to investigate the molecular epidemiological characteristics of the coxsackievirus A16 (CV-A16) that cause HFMD in Hangzhou, Zhejiang Province from 2021 to 2024. A total of 2,105-laboratory diagnosis HFMD cases were collected, of which 529 were CV-A16 positive samples. The detected number of CV-A16 began to rise in March, peaked in May–August, and then decreased in September. Its distribution was highest in children aged 1–5 years, with more males than females. The phylogenetic analysis indicated that the B1a and B1b subgenotypes were simultaneously prevalent in Hangzhou from 2021 to 2023. The B1c evolutionary branch detected in Hangzhou in 2023 experienced a sudden increase in quantity in 2024, with the same proportion as B1a, suggesting a change in the subgenotype prevalence of CV-A16 virus. Evolutionary analysis also confirmed that the CV-A16 in Hangzhou is genetically stable and the B1a lineage emerged earlier, followed by B1b, while B1c is a newly emerging lineage. There were three specific amino acid variations (P3S, I235V, and T240A) in the VP1 sequence of B1c. These findings have expanded the global genetic resources of CVA16 and provided supporting evidence for the prevention and control of HFMD. Meanwhile, the CV-A16 B1c outbreak branch that emerged in Hangzhou from 2023 to 2024 also highlights the necessity of strengthening the monitoring of virus evolution and epidemiological dynamics in this region.

## 1 Introduction

Hand, foot and mouth disease (HFMD), also known as hand, mouth and foot syndrome, is an acute febrile exanthematous infectious disease caused by enterovirus (EV) infections (Ventarola et al., 2015). The high-risk population is mainly infants and children, but it may also affect adults (Guerra et al., 2025). HFMD is mainly manifested as rash and herpes in the hands, feet, mouth and other parts of the body, as well as systemic fever. Some severe cases may be complicated by meningitis, encephalitis, acute flaccid paralysis, respiratory tract infection and myocarditis (Wong et al., 2010). HFMD was first reported in New Zealand in 1957 and has resulted in millions of attacks and several outbreaks worldwide (Nhu et al., 2021). And now this disease is prevalent worldwide, especially in the Asia-Pacific region, which not only imposed a huge burden on global healthcare, society and economy, but also has become a major public health issue worldwide (Ventarola et al., 2015; Zhuang et al., 2015). Meanwhile, following several large-scale outbreaks of hand-foot-mouth disease since 2008, the Ministry of Health of China and the World Health Organization have classified HFMD as a Class C notifiable infectious disease, and thus established a virological monitoring system for HFMD (Xing et al., 2014; Zhang et al., 2009).

Human EV is a non-enveloped single-stranded positive-sense RNA virus that has a complete genome length of approximately 7.4 kb, containing a large open reading frame (ORF) and 5′ untranslated region (5’UTR) and 3′ untranslated region (3’UTR) on both sides (Xie et al., 2024). The ORF encodes four structural proteins (VP1–VP4) and seven nonstructural proteins (2A–2C, 3A–3D), while the VP1 region nucleotide sequence is commonly used for genotyping EV that can be divided into four species (A–D) (Oberste et al., 1999). The majority of HFMD cases have been linked to EV-A, of which coxsackievirus A16 (CV-A16) and enterovirus A71 (EV-A71) are in most common (Zhang and Xu, 2013). Previously, the national HFMD monitoring data of China indicated that the main pathogen of HMFD in children was EV-A71. Since the launch of the inactivated EV-A71 vaccine in China in 2016, the number of HFMD caused by EV-A71 has decreased rapidly, while the proportion of CV-A16 has gradually increased (Hong et al., 2022). However, CV-A16 is also another major pathogen causing hand-foot-mouth disease in children. Due to rarely causes severe central nervous system-related diseases, it has not been thoroughly studied as EV-A71 has been.

CV-A16 is also a type of human EVs belonging to the *Enterovirus genus* of the *Picornaviridae* family. Since VP1 is an important marker for the genetic typing and evolutionary analysis of EV, CV-A16 can be initially classified into lineages A and B. The genotype B comprise sub-genotype B1 and B2. Furtherly, sub-genotype B1 can be categorized into three evolutionary branches, respectively B1a, B1b, B1c (Chu et al., 2024). Among them, the sub-genotype B2 first arose in Japan in 1981, but it has rarely appeared in the 21st century (Li et al., 2024). Currently, the sub-genotype B1 has been the most common since 1997, with B1a and B1b being first isolated in Japan in 1995 and 1998, respectively, (Zhang et al., 2010). It has dominated the global CVA16 epidemic for the past two decades, affected countries as follows China, Malaysia, Thailand, Vietnam and so on (Zhang et al., 2010). Notably, a new sub-genetic group B1c first appeared in Southeast Asia and some parts of Europe after 2000 (Xu et al., 2018; Hassel et al., 2017). However, there are relatively few cases of the B1c branch reported domestically, all of which are sporadic cases. In 2022, various cases relevant to B1c clades were reported in Beijing (Liang et al., 2024). Additionally, Zeng et al. showed that the emergence of the sub-genotype B1c has contributed to the prevalence of hand-foot-mouth disease in Guangdong region (Zeng et al., 2025). These clearly indicate that the sub-genotype B1c is on the rise.

HFMD has always been one of the diseases of global concern. The occurrence of its explosions around the world has become much more common, and it triggers large-scale epidemics every few years (Zhu et al., 2023). In China, the public health issue caused by HFMD has been very serious since 2007, with over 1 million new cases of HFMD occurring each year (Zhang et al., 2009; Hong et al., 2022). As a result, many provinces and cities have successively established HFMD case monitoring systems. The monitoring data from these systems consistently show that the incidence of CV-A16 infection is increasing every year (Li et al., 2024; Sun et al., 2017; Lv et al., 2025; Guo et al., 2022). In 2002, Zhejiang Province established an HFMD monitoring system. Over the past several decades, sporadic and outbreak cases of HFMD caused by human enteroviruses have been continuously reported (Sun et al., 2019). As an important central city in Zhejiang Province, Hangzhou is located in the southern wing of the Yangtze River Delta in China, with a permanent population of approximately 13 million. And it is an integral part of the Yangtze River Delta Urban Agglomeration, which serves as an international gateway for the Asia-Pacific region. Monitoring the epidemiological characteristics of HFMD in this area is of great significance for the control and prevention of HFMD. In this study, we conducted the whole genome sequence analysis of clinical samples of CV-A16 in Hangzhou from 2021 to 2024, clarifying its epidemiological and genetic characteristics. These research initiatives not only fill the gap in the literature regarding the genetic characteristics of CVA16 in the Hangzhou region but also provide a theoretical basis for the prevention and control of HFMD.

## 2 Materials and methods

### 2.1 Samples collection

The 13 district and county disease control and prevention centers (CDC) under the jurisdiction of Hangzhou City collected suspected HFMD case samples from sentinel hospitals in their respective areas. All laboratory-confirmed positive samples were sent to the Hangzhou City CDC laboratory for human EVs nucleic acid reconfirmation. During the period from 2021 to 2024, a total of 2,105 laboratory diagnosis HFMD cases were collected. Among them, 676 laboratory diagnosis HFMD cases were collected in 2021, 397 in 2022, 546 in 2023, and 486 in 2024. Most of the cases had mild symptoms, including fever, rash, oral inflammation, oral herpes, oral ulcers, nausea, vomiting, coughing, and runny nose. The samples were collected in specialized virus sampling tubes and sent to the laboratory for storage at −80 °C in the refrigerator. During the sample collection process, patient consent was obtained.

The data of positive cases submitted by the 13 district and county CDCs included information such as gender, age and onset time. These data were sorted using Excel software. Statistical analysis was conducted to describe the epidemiological characteristics by SPSS v26.0, including demographic characteristics, gender and age distribution, as well as seasonal variations. The chi-square test was used to evaluate the correlations among the demographic variables. The *p* < 0.05 was set as the cutoff value for statistical significance.

### 2.2 Virus RNA extraction and identification of EV

Viral RNA was extracted from clinical specimens using the QIAamp Viral RNA Mini Kit (Qiagen, Germany) according to the manufacturer’s instructions. The extracted viral nucleic acids were suspended in 50 μL of water without nuclease and stored at −80 °C until use. Each sample was tested for nucleic acids using QuantStudio Real-time Fluorescence Quantitative PCR System (Applied Biosystems, United States). And the 3-channel (EV-A71, CV-A16, and pan-EV) real-time fluorescence RT-PCR detection kit (Jiangsu Bioperfectus Technologies, China) was adopted for genotyping. The detection procedures of RT-PCR and reagent systems were all set up in accordance with the instructions of the reagent kit.

### 2.3 Whole-genome sequencing of CV-A16

The whole genome sequencing of CV-A16 was performed using MinION nanopore sequencing. Samples for sequencing were selected from the annual CV-A16 positive samples based on the following criteria: 1. *Ct*≤25; 2. Covering 13 districts and counties of Hangzhou City; 3. Cover the months when CV-A16 was prevalent. A total of 221 positive samples of CV-A16 were selected for whole genome sequencing. The information of sequencing samples in this study was all recorded in Supplementary Table S1. According to the product manual, high-quality cDNA of CV-A16 virus was captured using EV whole genome sequence kit (Beijing Applied Biological Technologies, China). The libraries for MinION sequencing were prepared using Nanopore sequencing kit (Oxford Nanopore Technologies (ONT), United Kingdom) SQK-RBK114.96, mainly including amplicons purification, adaptor connection, fragmentation. The quantified libraries were loaded in MinION flow cells (MIN114) after the quality control (QC) was tested using ONT MinKNOW software. The flow cells then ran on the GridION X5 until production of reads slowed. The raw sequencing data after disembarking was imported into Geneious Prime v 2024.0 for analysis. The trimmed sequence reads were assembled based on mapped to reference sequences method. PQ182728 was used as reference for CV-A16 strain. Meanwhile, the consensus sequences were drawn for the subsequent analysis.

### 2.4 Phylogenetic analysis of CVA16

Based on the 61 reference sequences of CV-A16 downloaded from the GenBank database, the phylogenetic relationship between the CV-A16 sequence in this study and other CV-A16 strains was analyzed, and the genetic group of the CV-A16 sequence in this study was identified. And the relevant information of the reference sequences was recorded in Supplementary Table S2. The identity of consensus sequences was determined using the BLAST search tool.^1^ We constructed maximum likelihood phylogenetic trees with IQ-TREE v3.0.1, using ModelFinder to find the best nucleotide substitution mode (Trifinopoulos et al., 2016; Nguyen et al., 2015). The reliability of sequence clusters was evaluated with SH-aLRT (1,000 replicates) and UFBoot2 (10,000 replicates) (Wiegert et al., 2024). The online visual tool iTOL^2^ was used to visualize the phylogenetic tree of CV-A16.

### 2.5 Evolutionary analysis

In order to analyze the nucleotide consistency, the nucleotide entropy values of each gene fragment of CV-A16 in Hangzhou calculated by the biopython package. Based on the Regression of root-to-tip distances in the Treetime software, the evolutionary rates of each gene fragment of CV-A16 in Hangzhou were further analyzed (Sagulenko et al., 2018). The time-scaled tree and historical population dynamics including time of the most recent common ancestor (tMRCA) and the rate of evolution (substitution/site/year, s/s/y) were estimated using BEAST package v1.10.4 with a Bayesian Markov chain Monte Carlo (MCMC) approach for phylogenetic reconstruction (Suchard et al., 2018). And the Bayesian Skyline model with strict molecular clock was employed. The grid structure has been completed, and the annual population size has been estimated. The MCMC lengths of 400,000,000 generations with sampling every 4,000 generations were performed and individually obtained the most effective sample sizes over 200 traced in Tracer v1.7.1. The maximum clade credibility (MCC) tree was determined using TreeAnnotator v1.10.4 and visualized in tool iTOL (see text footnote 2, respectively). The tMRCA and its 95% highest probability density (95% HPD) were expressed as a year.

### 2.6 Recombination analysis

Based on the 5’ UTR, P1, P2 and P3 regions, the Geneious software was used to compare all subtypes of CV-A16 in Hangzhou with the prototype strain EV-A. The sequences of the prototype strain were obtained from GenBank. One random sequence was selected from each subtype of CV-A16 in Hangzhou. The potential recombination events were analyzed using the SimPlot software (version 3.5.1). In Simplot, the Kimura two-parameter model was used to execute the nucleotide substitution model, and the ratio of nucleotide transitions and transversions was set to 2.0 (Yang et al., 2025).

## 3 Results

### 3.1 The prevalence of CV-A16 in Hangzhou from 2021 to 2024

**Figure 1:**
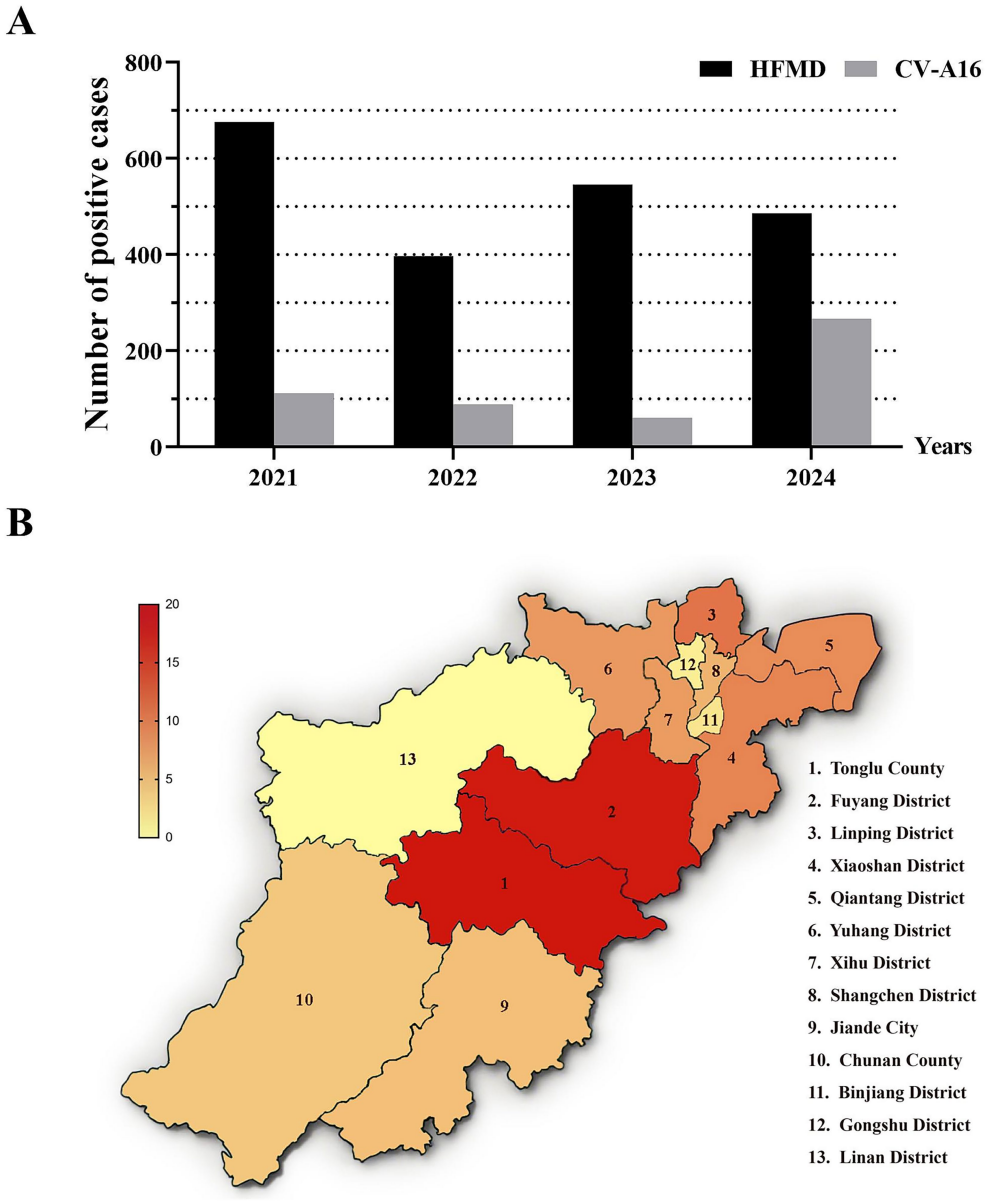
**(A)** Yearly distribution of laboratory-diagnosed cases of HFMD and CV-A16 during 2021–2024 in Hangzhou, China. **(B)** The percentage of laboratory-diagnosed CV-A16 cases in each district and county of Hangzhou City from 2021 to 2024. The shade of the color is used to indicate the proportion of CV-A16.

According to the data of laboratory-diagnosed HFMD samples sent from all districts and counties of Hangzhou, 676 laboratory-diagnosed HFMD samples were sent in 2021, 397 laboratory-diagnosed HFMD samples were sent in 2022, 546 laboratory-diagnosed HFMD samples were sent in 2023, and 486 laboratory-diagnosed HFMD samples were sent in 2024 (Figure 1A). Among them, the number of CV-A16 positive samples was 112 (16.57%) in 2021, 89 (22.42%) in 2022, 61 (11.17%) in 2023, and 267 (54.94%) in 2024 (Figure 1A). It can be seen that the number of CV-A16 fluctuated little from 2021 to 2022, but decreased in 2023 and then increased significantly in 2024. These data indicate that the detection rate of CV-A16 in Hangzhou fluctuates.

By analyzing the data of laboratory-diagnosed CV-A16 samples sent by each district and county in Hangzhou, it was found that the average number of CV-A16 in each district from 2021 to 2024 was 40. The distribution pattern of CV-A16 in Hangzhou was higher in the northeast and lower in the other parts of Hangzhou (Figure 1B). Among all the areas studied, Tonglu County and Fuyang District had the highest number of CV-A16, about 2–3 times the average number, while Binjiang District, Gongshu District and Linan District had the lowest number of CV-A16 samples, which was only ≤ 5%.

### 3.2 Seasonal characteristics of CV-A16 in Hangzhou from 2021 to 2024

**Figure 2:**
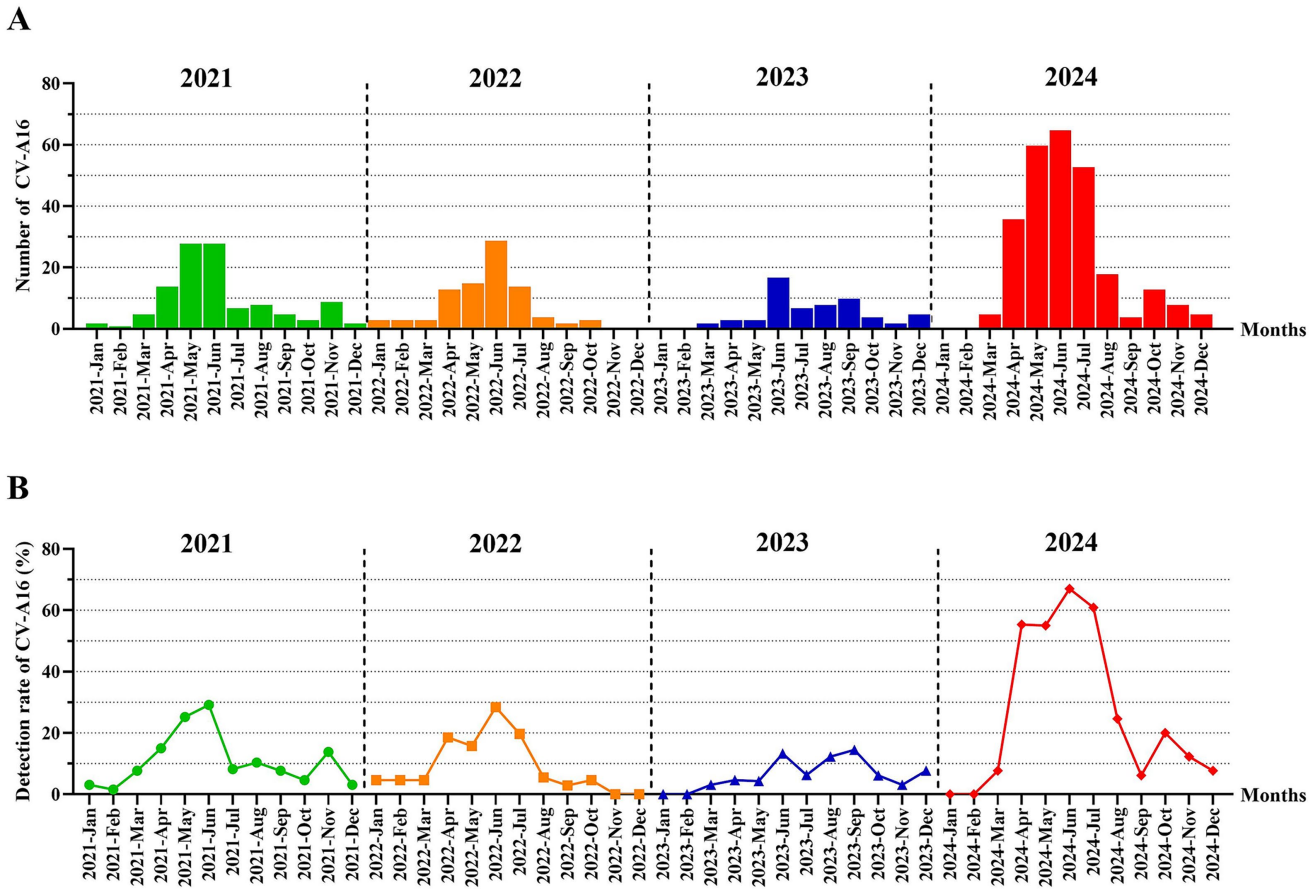
**(A)** The number distribution of laboratory-diagnosed CV-A16 cases in Hangzhou by month between 2021 and 2024. **(B)** The monthly detection rate distribution of CV-A16 cases confirmed by laboratories in Hangzhou during the period from 2021 to 2024.

The numbers of laboratory-diagnosed CV-A16 in Hangzhou from 2021 to 2024 has an obvious seasonal pattern (Figure 2). From the trend of the number of CV-A16, it could be inferred that the number of CV-A16 increased from March, and began to be prevalent from May to August, reached the peak in June, and decreased in September at last every year. The peak period of the epidemic was from May to August, which coincided with the season of fecal-oral transmitted diseases (usually summer), and then decreased in the later months with small fluctuations. However, in terms of years, the most obvious change in regularity was observed in 2024 compared to 2021–2023.

### 3.3 Distribution of CV-A16 by gender and age in Hangzhou, between 2021 and 2024

**Figure 3:**
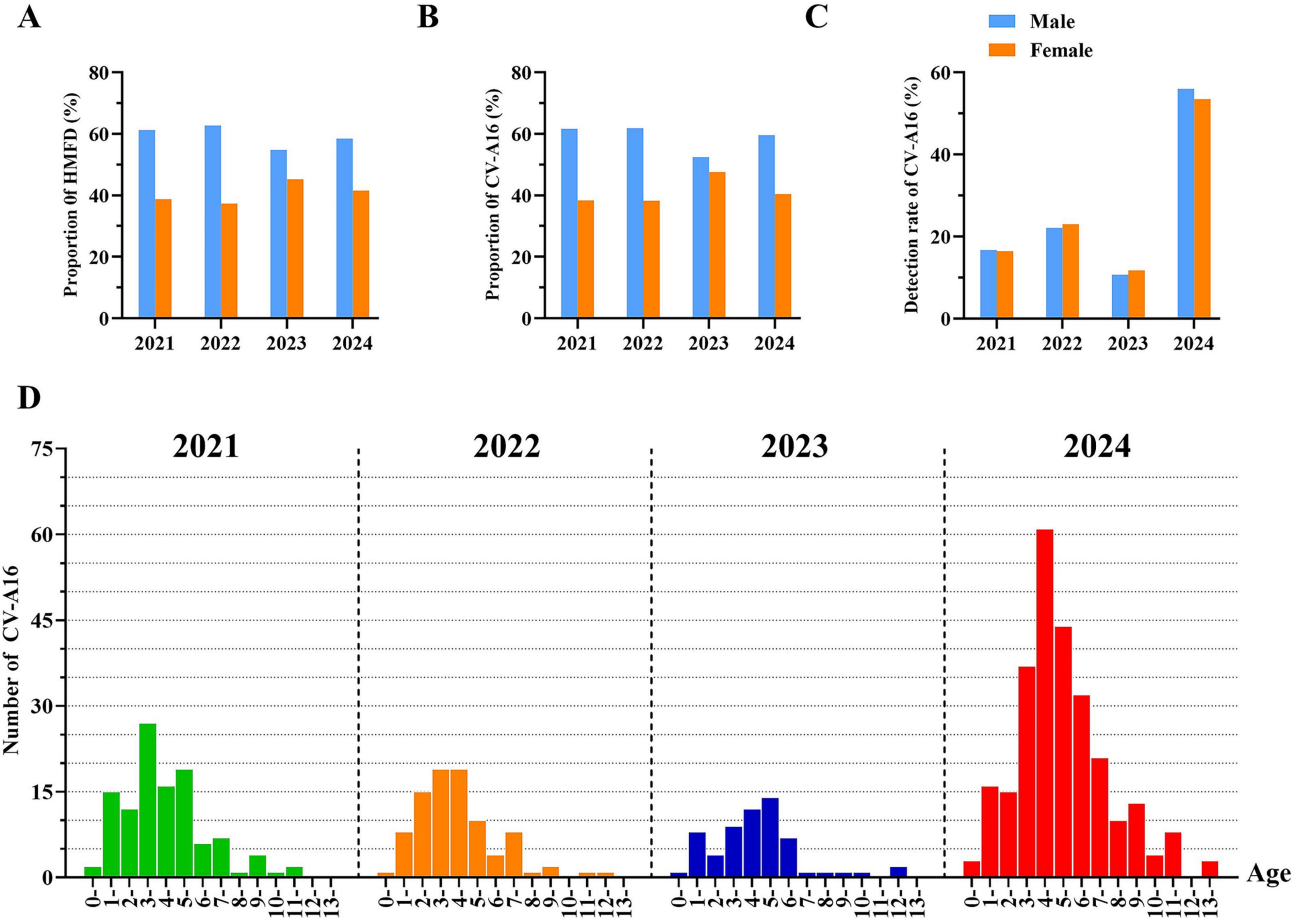
**(A)** The proportion of laboratory-diagnosed HFMD cases in Hangzhou between 2021 and 2024 by gender. **(B)** The proportion of laboratory-diagnosed CV-A16 cases between 2021 and 2024 by gender. **(C)** Detection rate of laboratory-diagnosed CV-A16 cases in Hangzhou between 2021 and 2024 by gender. **(D)** Age distribution of l laboratory-diagnosed CV-A16 cases in Hangzhou city, 2021–2024. Categorize the whole population into the following groups: 0–1, 1–2, 2–3, 3–4, 4–5, 5–6, 6–7, 7–8, 8–9, 10–11, 11–12, 12–13 and over 13 years old. The four colors represent different years, and the height of the bar graph represents the corresponding number of cases.

During the period from 2021 to 2024, the number of laboratory-confirmed positive HMFD and CV-A16 among males was consistently higher than that among females for four consecutive years (Figures 3A,B). Specifically, from 2021 to 2024, The proportion of male samples is approximately 60%, while that of female samples is approximately 40%. Among the total number of positive HFMD cases in male, the detection rate of CV-A16 males was 16,67% in 2021, 22.09% in 2022, 10.70% in 2023, and 55.99% in 2024. Among the total number of positive HFMD cases in female, the detection rate of CV-A16 female was 16.41% in 2021, 22.97% in 2022, 11.74% in 2023, and 53.47% in 2024 (Figure 3C).

The laboratory-diagnosed cases of CV-A16 in Hangzhou, Zhejiang Province from 2021 to 2024 was the highest among children aged 1–5 years old, followed by those aged 6–7 years old. The samples remained above 70% for four consecutive years, and the majority of the positive CV-A16 were scattered children and preschool children. There was a statistically significant difference among CV-A16 positive samples of different age groups (*p* < 0.05). The peak occurrence was observed in the 3–5 age group and it persisted at over 50% for 4 years, suggesting that children in this age range might be highly susceptible to CV-A16 (Figure 3D). And within the 3–9 age group, the number of laboratory-diagnosed CV-A16 in 2024 was obviously higher than in other years, and this difference was statistically significant (*p* < 0.05).

### 3.4 Phylogenetic analysis of CV-A16

**Figure 4:**
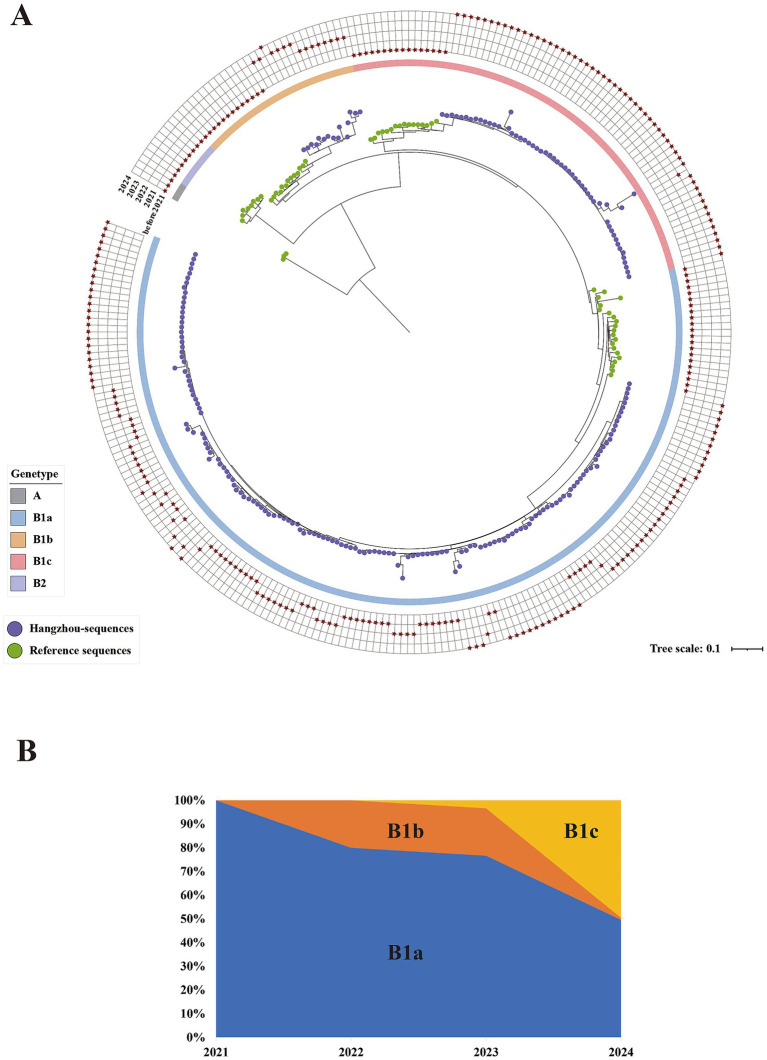
**(A)** Maximum likelihood tree constructed based on the 282 complete genome sequences of CV-A16 in Hangzhou from 2021 to 2024. The outer grid represents CV-A16 sequences from different years, with the grids from the inside out representing the years 2021, 2022, 2023, and 2024, and marked with asterisks. The inner circle represents different gene clusters/sub-gene clusters, labeled with different colors. The ends of each leaf are represented by circles of different colors for the reference sequences and the sequences from Hangzhou. The green circles represent the references sequence, and the purple circles represent the Hangzhou sequences. **(B)** The temporal distribution of CVA16 subgenotypes (B1a, B1b and B1c) in Hangzhou 2021–2024.

Based on the whole genome, a phylogenetic tree analysis was conducted on 221 laboratory-diagnosed CV-A16 samples from Hangzhou during the period from 2021 to 2024, as well as the relevant genotypes of CVA16 in the GenBank database. Over the past 4 years, CV-A16 of Hangzhou has been classified into three branches based on the diversity of the VP1 sequence, all belonging to the B1 sub-gene group, namely B1a, B1b, and B1c (Figure 4A). We found that the distribution patterns of the subgenotype B1a and B1b varied significantly among different years. Among them, the annual distribution of the B1a subgenotype was more widespread, starting from 2021 and continuing until 2024. The number detected by this subgenotype has always accounted for the major portion. On the contrary, the proportion of the B1b subgenotype over the 4 years was not high. Similarly, the subgenotypes B1a and B1b showed a continuous decline in the number of detections starting from 2021(Figure 4B). Moreover, we noticed that in the Hangzhou area, a relatively rare B1c subgenotype was detected in 2023, and the detection rate then showed an upward trend. In the selected sequencing samples for 2024, the B1c subtype accounted for 49.54%. The similarity analysis revealed the following homology situations: The nucleotide similarity of B1a and B1b was 81.21–95.42%; the similarity of B1a and B1c was 84.03–91.59%; the similarity of B1b and B1c was 78.35–95.42%. Among the clinical samples sequenced in 2024, the B1c subgenotype samples showed a full genome similarity of 87.63–96.78% to the early prevalent strains in France and other Asian regions. These sequenced samples had the highest sequence consistency with the isolates from India (GenBank accession number MH780757.1). Furthermore, the ancestral reconstruction results indicate that the B1c subgenotype differs from the B1a and B1b subgenotype in terms of specific mutations at positions 3 (P3S), 213(A213E), 215(P215L), 217(S217A), 235 (I235V), and 240 (T240A) of the VP1 protein.

### 3.5 Analysis of evolution

**Figure 5:**
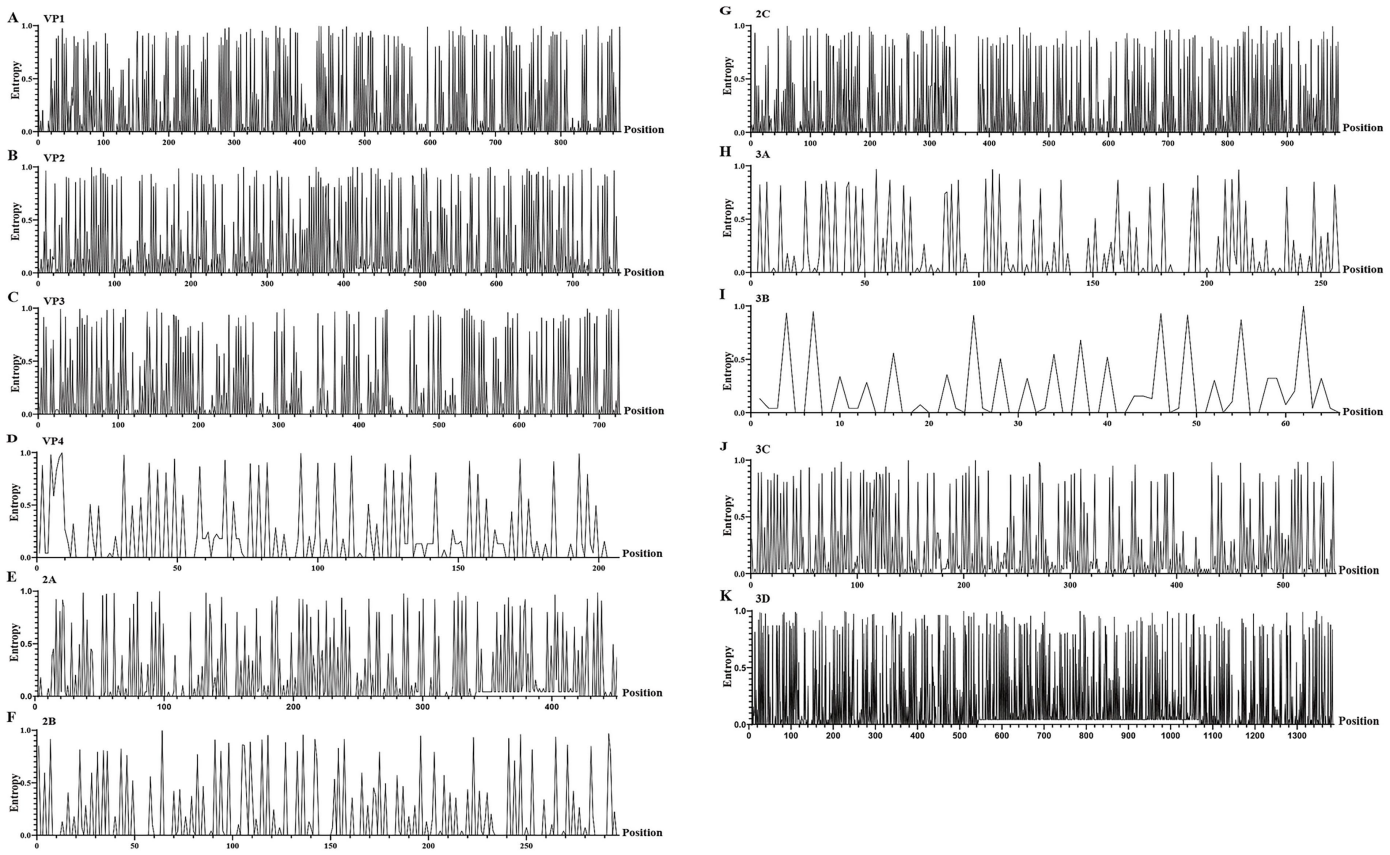
The entropy value of nucleotide sites for different gene segments of CV-A16. **(A–K)** represent the diagrams of entropy of the nucleic acid sites of the VP1, VP2, VP3, VP4, 2A, 2B, 2C, 3A, 3B, 3C and 3D, respectively.

Through the analysis of nucleotide entropy values of the whole genome sequence, 37.12% of the sites in the P1 coding region of CV-A16 in Hangzhou from 2021 to 2024 showed non-consistency (Figures 5A–D). Among them, the highest number of sites with nucleotide entropy values is VP1 gene (Figure 5A). In the P2 coding region, 37.14% of the sites exhibit non-consistency (Figures 5E–G). Among them, the highest number of sites with nucleotide entropy values is 2C gene (Figure 5G). In the P3 coding region, 56.44% of the sites have non-consistency (Figures 5H–K). Among them, the highest number of sites with nucleotide entropy values is 3D gene (Figure 5K). The analysis results of Tree Time show that the P1 coding region is the least stable, and the VP1 gene segment within it is more prone to mutations, while the P2 coding region is relatively conservative (Table 1).

**Table 1:** The genetic characteristics of each gene of CV-A16 in Hangzhou from 2021 to 2024.

Gene	Evolution rate (10^−3^)	Root date (year)
VP1	3.08 ± 0.30	1978.0 ± 5.11
VP2	2.06 ± 0.30	1993.2 ± 5.47
VP3	2.45 ± 0.40	1996.5 ± 4.19
VP4	1.82 ± 1.00	1988.5 ± 20.77
2A	1.48 ± 0.50	1972.0 ± 18.66
2B	1.76 ± 0.80	1976.1 ± 22.35
2C	0.90 ± 0.30	1878.6 ± 45.60
3A	2.41 ± 0.90	2014.6 ± 3.00
3B	2.02 ± 3.00	1987.6 ± 57.04
3C	1.26 ± 0.50	1923.4 ± 36.49
3D	1.56 ± 0.20	2003.6 ± 2.55

**Figure 6:**
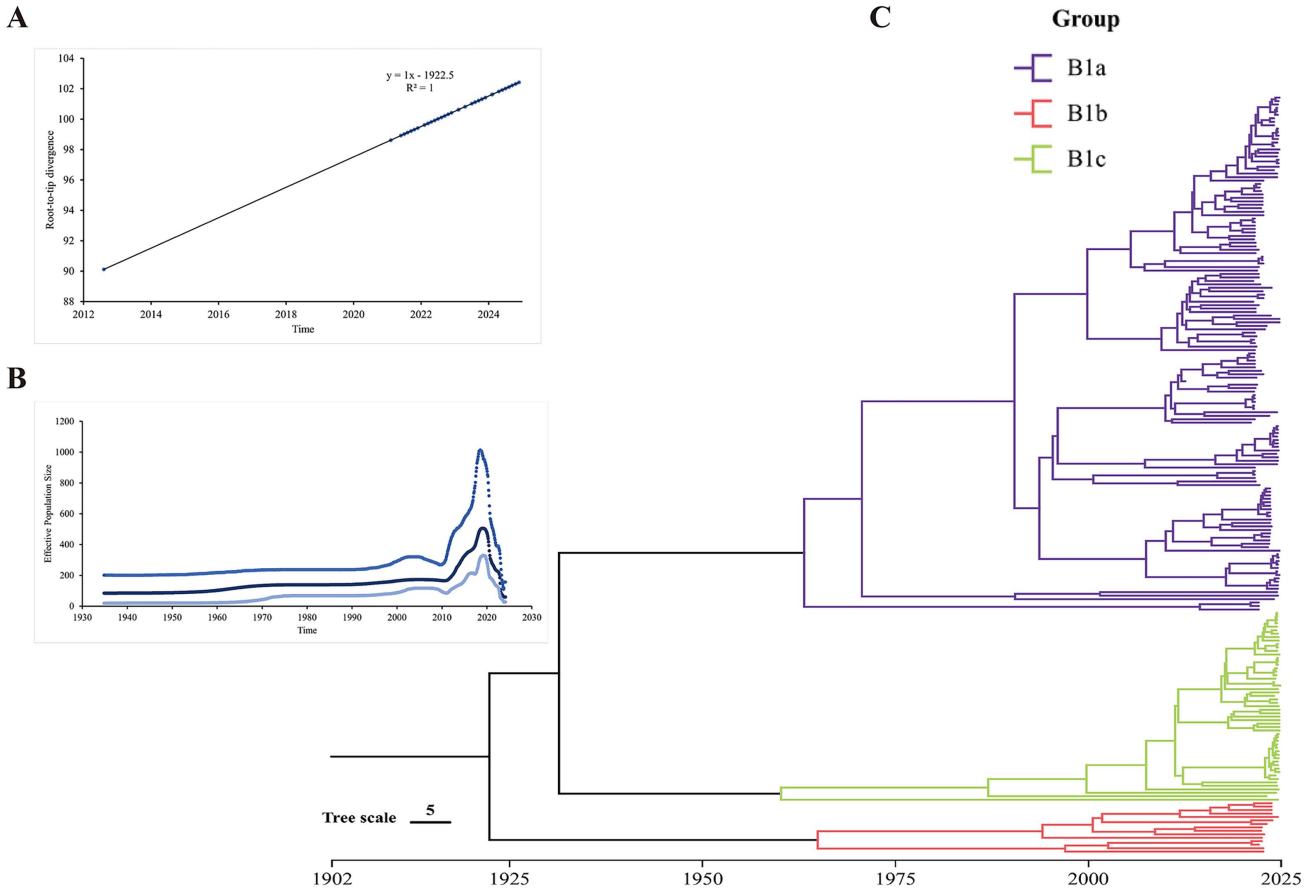
Molecular clock for evolutionary analysis of CV-A16 genotype B1 based on the whole genome sequence. **(A)** Evolutionary time signal through root-to-tip divergence analysis. **(B)** Population history of CV-A16 genotype B1 inferred by Bayesian skyline model. The x-axis represents the time scale in years, and the y-axis represents the effective population size. **(C)** Bayesian Maximum Evolutionary Branch Confidence (MCC) phylogenetic tree.

The root-to-tip analysis indicated that CV-A16 has a positive time signal (Figure 6A). The estimated effective population dynamics over time using Bayesian skyline analysis showed that the significant fluctuations of CVA16 occurred between 2010 and 2020 and it started to rise in 2010 and then declined after 2020 (Figure 6B). The time-scale MCC tree indicated that the sub-genotype B1a and B1c of CV-A16 in Hangzhou first differentiated around 1933. Among them, B1a was divided into two different branches around 1962. Both of these branches include the sequences from 2021 to 2024. Similarly, B1b was divided into two branches around 1964 (Figure 6C). The tMRCA of CV-A16 sub-genotype B1a can be traced back to 1962.5. The tMRCA of CV-A16 sub-genotype B1b can be traced back to 1964.2. The tMRCA of CV-A16 sub-genotype B1c can be traced back to 1959.4 (Table 2). We can observe that three evolutionary branches have stabilized in Hangzhou. Among them, B1a was prevalent earlier, and B1b was constantly circulating with B1a from 2000 onwards. However, the position of the nodes in the B1c branch is closer to the recent period, indicating that this branch has only recently become prevalent.

**Table 2:** The mean time of the most recent common ancestor (tMRCA), 95% highest probability density (HPD) and estimated the mean substitution rate of Hangzhou CVA16.

Virus	Subgenotype	tMRCA	95% HPD interval	Mean rate (10^−3^ s/s/y)	95% HPD interval (10^−3^ s/s/y)
Coxsackievirus A16	B1a	1962.5	1938.5–1977.1	0.926	0.781–1.05
	B1b	1964.2	1952.4–1974.1	0.926	0.781–1.05
	B1c	1959.4	1947.3–1969.1	0.926	0.781–1.05

### 3.6 Recombination analysis

In this study, one representative sample of each of the CV-A16 B1a, B1b, and B1c types was selected for the presentation of the recombination results. Similarity and BootScan analysis showed that the B1a and B1c of CV-A16 was similar to CV-A8 in the 5′-UTR region, and similar to the CV-A16 prototype strain in the P1 region (Supplementary Figures S1A–C). The P2 and P3 regions were similar to EV-A71, CV-A3, CV-A12, and CV-A8 at different sites (Supplementary Figures S1A–C). But the B1b of CV-A16 was similar to CV-A4 in the 5′-UTR region, and were also similar to the CV-A16 prototype strain in the P1 region. Additionally, it was also similar to EV-A71, CV-A3, CV-A12, and CV-A8 in the P2 and P3 regions (Supplementary Figure S1B). Although there were some crossover phenomena at the sites, the Simplot software did not indicate that any significant recombination events occurred in the CV-A16 sequence used in this study.

## 4 Discussion

Human EVs are a group of highly contagious viruses that can cause various diseases, among which HFMD is on (Tapparel et al., 2013). The typing and monitoring of EVs are crucial for tracking epidemiological trends and potentially linking them to clinical manifestations. At present, more than one hundred different EV genotypes have been identified worldwide, and some of these infections can lead to severe morbidity and mortality rates (Brouwer et al., 2021). Over the years, different countries have used various methods to detect and classify EV in disease monitoring studies (Faleye et al., 2016). Due to the extensive genetic diversity of enteroviruses, among which there are 23 variants of Group A enteroviruses (EV-A), traditional targeted techniques for genetic typing and research may become challenging (Wong et al., 2010; Kim et al., 2018). Therefore, for clinical samples, most studies have been limited to molecular characteristic analysis based on the VP1 nucleotide sequence of enteroviruses or to conducting whole-genome characteristic analysis through cell culture of clinical samples (Yuan et al., 2024; Sun et al., 2023; Pattassery et al., 2023). In this study, we directly captured viral nucleic acids from clinical samples and obtained the nearly 7,400 bp full genome sequence of CV-A16 through amplicon sequencing. As a supplement to traditional research methods, direct whole-genome sequencing of clinical samples can efficiently complete the molecular typing of enteroviruses and also provides a more comprehensive phylogenetic analysis.

Based on the positive monitoring data of CV-A16 in Hangzhou from 2021 to 2024, this study provides an overview of the general epidemiological situation of CV-A16 in Hangzhou. The results show that children aged 1–5 years old account for the majority of the outbreaks, which is similar to the HFMD reports in other regions of China and other countries (Yin et al., 2018; Chen et al., 2024). A maternal–maternal-child matching study in Jiangsu, China, found that the average positive rate of maternal antibodies at birth was 90% (Zhu et al., 2012). This indicates that children under 1 year old usually have maternal antibody protection, which may result in a lower incidence of CV-A16 infection. Additionally, as maternal antibodies naturally decline over time, the antibody levels of newborns gradually decrease from birth to 1 year old. Li et al. (2021) demonstrated that the positive rate of CV-A16 antibodies gradually increased from 1 to 5 years old, and could only remain at a relatively high level after the age of 5. Children aged 1–5 have an immature immune system compared to older age groups and their healthy habits have not been fully developed, making them more susceptible to infections from various EVs. Therefore, this age group should be the key target for controlling and preventing CV-A16. This study also indicated that the number of laboratory-confirmed CV-A16 has consistently been higher in men than in female. Although there is no significant difference, this trend has persisted for 4 years. Despite the phenomenon of higher risk of mild and severe HFMD (with an OR range of 1.2 to 2) in men showed by most studies (Koh et al., 2016), a few studies have indicated that there is no significant difference in the infection rate of EVs between men and female (Chang et al., 2002; Ang et al., 2011). On account of little research on the transmission of EVs between genders, we can only make inferences based on previous studies. It is assumed that boys are inclined to be more vivaciously compared to girls and excessive outdoor activities allow them to come into more frequent contact with unclean toys and facilities, which increases the exposure level. Additionally, the differences in host immune status between men and female may also be a cause for this gender difference (Xing et al., 2014; Lv et al., 2025).

Overall, the number of laboratory-confirmed CV-A16 in Hangzhou showed seasonal characteristics, which is consistent with the results reported in other cities in China (Yin et al., 2018; Yang et al., 2023). The number of laboratory-confirmed CV-A16 in Hangzhou increases sporadically from January to March each year, rises again in April, reaches the main epidemic peak from May to August, and then has a secondary peak in autumn (from October to November; Figure 2). Many studies have found that climatic factors, such as sunshine duration, temperature, precipitation, and air pressure, are the main predictors of the epidemiology of HFMD in the region (Hii et al., 2011; Wang et al., 2011). For example, in Japan, the number of hand-foot-mouth disease cases increases with the increase in average temperature and humidity (Koh et al., 2016). In addition, other factors, such as school holidays or the start of the school term, may also be reasons for the seasonality (Wang et al., 2016). At the same time, through the statistics of the number of CV-A16 in each county and district, we found that the number of CV-A16 positive cases in Fu Yang County and Tong Lu County were at a high level. Due to Hangzhou being in a subtropical monsoon climate, along with significant differences in population among the counties and districts and differences in local medical levels, this may cause the transmission and replication ability of CV-A16 to vary in different regions. The above epidemic pattern of CV-A16 alerts that during the peak epidemic season, scientific prevention and control measures should be implemented, and local conditions should be taken into account to effectively reduce the incidence rate.

The alternation of outbreaks of CVA16 and EV-A71, which are considered the main pathogens of HFMD, has persisted in Southeast Asia for over a decade (Li et al., 2005; Iwai et al., 2009). After vaccination with the inactivated EV-A71 vaccine, the pathogen spectrum of HFMD has undergone certain changes, with the number of cases caused by EV-A71 gradually decreasing. However, CVA16 is still prevalent. In several cities in China, such as Taiyuan, Yantai, and Shenyang, the cases of HFMD caused by CV-A16 have shown a significant upward trend and CV-A16 has become the main pathogen according to relevant monitoring data (Li et al., 2024; Guo et al., 2022; Yuan et al., 2024; Sun et al., 2023). Therefore, we focused on studying the relevant information of CV-A16 in order to enhance our understanding of this pathogen. In this study, we counted the number of laboratory-confirmed HFMD and CV-A16 submitted by the districts and counties from 2021 to 2024. The research found that the number of laboratory-confirmed HFMD in 2021 was higher than in other years. We suspect that this is related to the prevention and control policies implemented during the novel coronavirus disease 2019 (COVID-19) pandemic in Hangzhou. However, the number of laboratory-confirmed CV-A16 did not vary much between 2021 and 2022, and even decreased in 2023. Researches on HFMD indicates that the prevalence of COVID-19 in 2019 significantly affected the incidence of HFMD (Lv et al., 2025; Zhou et al., 2023). Over the successive years, people have developed good habits through measures such as self-isolation, maintaining social distance, wearing masks, and frequently keeping hands clean. These strong intervention measures have helped control the infection of CV-A16 (Li et al., 2024). Surprisingly, the number of CV-A16 in 2024 increased significantly, and its B1c subgenotype also grew rapidly that year. This led to the speculation that the B1c subtype might have been the main driving factor for the surge of CV-A16 in Hangzhou. Similar results were also observed in Guangdong (Zeng et al., 2025).

Up to now, CV-A16 related to HFMD has been prevalent in Southeast Asia and the Pacific region for several decades (Mao et al., 2014). Based on the phylogenetic tree and genetic diversity of the VP1 gene, global CV-A16 strains can be classified into three genotypes: A, B, and D (Oberste et al., 1999). Genotype B can be further divided into three subtypes. In recent years, the subgenotype B1 has been the most prevalent worldwide (Fu et al., 2020). Subgenotype B1 is composed of three evolutionary branches: B1a, B1b, and B1c. Evolutionary clade B1a was first reported in Japan in 1995 and subsequently became the predominant strain in Chinese Mainland, Malaysia, and Thailand (Yi et al., 2021). Since the 21st century, B1a and B1b strains have co-circulated in China, especially in the eastern region (Lv et al., 2025). These two evolutionary branches are the main CV-A16 subgenotypes involved in HFMD cases (Sun et al., 2014). Similarly, from the molecular clock timeline of this study, it can also be inferred that the three branches in the Hangzhou area have different evolutionary histories. The B1a branch may be the oldest, with strong environmental adaptability and continuous transmission capacity, and it may have formed endemicity in Hangzhou and even a wider area; the B1b branch comes next, while the B1c branch is a relatively new and recently active lineage. This phenomenon of multiple lineages co-circulating indicates that the CV-A16 virus in Hangzhou has rich genetic diversity. This suggests that the source of the virus is multiple, possibly involving the continuous evolution of local viruses and the repeated introduction of new external virus strains. In previous reports, the B1a and B1b subgroups have frequently shown a cycle pattern of appearance, disappearance, and re-appearing in different regions of China (Li et al., 2024). Similar situations also occurred in this study. Additionally, the evolutionary branch B1c first appeared in Malaysia and was subsequently discovered in multiple countries such as Russia, France, India, and Japan (Chen et al., 2013). In 2013, it triggered a HFMD outbreak in India (Ganorkar et al., 2017). In recent years, B1c series virus strains have occasionally been found in Thailand (2023) (Taoma et al., 2025) and Palestine (2024) (Dumaidi et al., 2025). Reports on the B1c branch within China are limited to individual cases, including cases in Shenyang (2017, 2022) (Li et al., 2024), Guangzhou (2018) (Xie et al., 2020), and Beijing (2022) (Liang et al., 2024). By sequencing analysis, we detected the rare B1c clade in 2023, and the B1c clade was on par with the B1a subtype in 2024, suggesting that there may have been a genotype transfer of CV-A16 in Hangzhou. Interestingly, the number of CV-A16 positive cases in 2024 increased significantly compared to the previous 3 years, which further suggests that the emergence of the B1c subtype contributed to the prevalence of CV-A16 in 2024. However, due to the lack of the latest molecular monitoring data from other regions in China, we suspect that the emergence of the new lineage B1c may indicate a new evolutionary or introduction event. The underlying driving factors might be related to the its own adaptive mutations, changes in population immunity, or travel and migration. Furthermore, the research found that the VP1 gene had the highest evolutionary rate among all the gene segments, which might have contributed to the evolution of the lineage. By comparing the VP1 coding sequences of B1a, B1b and B1c, three primary amino acid mutations were identified: P3S, I235V, and T240A. In recent domestic studies on CVA16, these three mutations have also been reported (Liang et al., 2024; Hu et al., 2021).

EVs can undergo extensive genetic recombination, resulting in genetic variations and evolution (Liu et al., 2014). Recombination is one of the main mechanisms of evolution for enteroviruses. However, the CV-A16 has not undergone multiple recombination events with other EV-A donor sequences currently. In the evolutionary analysis, the evolutionary rates of the various gene fragments and three lineages of CV-A16 ranged from 0.90–3.08 × 10^–3^ substitutions/site/year. Other studies on the evolutionary analysis of CV-A16 indicate that the evolutionary rate of CV-A16 is 3.36–6.66 × 10^−3^ substitutions/site/year (Zhao et al., 2016; Han et al., 2020). It can be seen that CV-A16 in Hangzhou is possibly in a relatively stable period of evolution. Furthermore, some studies have shown that recombination events tend to occur more frequently in specific years and regions, possibly indicating a local outbreak and spread of one or several independent and successful recombinant strains (Liu et al., 2014; Rao et al., 2017). Moreover, this study merely represents the epidemic trend of CV-A16 in the Hangzhou during the period from 2021 to 2024. China has a vast territory, and the dominant genotypes or lineages that are prevalent in different regions may vary. The opportunities for co-circulation and recombination with other enteroviruses (such as CV-A4, CV-A8, EV-A71, etc.) also differ. It given that it may be in a relatively stable evolutionary period, and new recombination events may not be captured.

## 5 Conclusion

The study related to CV-A16 elucidated the molecular epidemiological characteristics in Hangzhou, Zhejiang Province from 2021 to 2024. The CV-A16 in Hangzhou is affiliated with factors such as month, gender, age and region, and there are certain differences. The main lineage of CV-A16 in Hangzhou is the B1 subtype, and the relatively rare B1c strain was widely present in 2024, which might be associated with the significant increase in the number of CV-A16 infections in 2024. Overall, this study provides valuable insights into the epidemiological pattern of CV-A16 in the population of Hangzhou, further deepening our comprehensive understanding of CV-A16. Our research results also provide valuable information for the prevention and control of HFMD.

## Data Availability

The datasets presented in this study can be found in online repositories. The names of the repository/repositories and accession number(s) can be found in the article/Supplementary material.

## Footnotes

^1^ http://www.ncbi.nlm.nih.gov/blast

^2^ https://itol.embl.de/
