# Supplementary material for: Epidemiology and genetic characteristics of coxsackievirus A16 associated with hand-foot-mouth disease in Hangzhou city, Zhejiang province from 2021 to 2024
Source: Front Microbiol. 2025 Nov 14;16:1698485. doi: 10.3389/fmicb.2025.1698485 (PMC12660211; doi:10.3389/fmicb.2025.1698485)
Supplement: Supplementary file 1 [file Table_1.docx]

**Table S1. Information about the 221 Sequencing samples of CV-A16 in this study**

| **Sample name** | **Separate area** | **Sampling Year** | **Specimen type** | **genotyping** | **GenBank accession** |
| --- | --- | --- | --- | --- | --- |
| 2021CV16-HZ-01 | Hangzhou | 2021 | Throat swab | CVA16(B1a) | PX448819 |
| 2021CV16-HZ-02 | Hangzhou | 2021 | Throat swab | CVA16(B1a) | PX448821 |
| 2021CV16-HZ-03 | Hangzhou | 2021 | Throat swab | CVA16(B1a) | PX448822 |
| 2021CV16-HZ-04 | Hangzhou | 2021 | Throat swab | CVA16(B1a) | PX448918 |
| 2021CV16-HZ-05 | Hangzhou | 2021 | Throat swab | CVA16(B1a) | PX448892 |
| 2021CV16-HZ-06 | Hangzhou | 2021 | Throat swab | CVA16(B1a) | PX448893 |
| 2021CV16-HZ-07 | Hangzhou | 2021 | Throat swab | CVA16(B1a) | PX448906 |
| 2021CV16-HZ-08 | Hangzhou | 2021 | Throat swab | CVA16(B1a) | PX448913 |
| 2021CV16-HZ-09 | Hangzhou | 2021 | Throat swab | CVA16(B1a) | PX448834 |
| 2021CV16-HZ-10 | Hangzhou | 2021 | Throat swab | CVA16(B1a) | PX448838 |
| 2021CV16-HZ-11 | Hangzhou | 2021 | Throat swab | CVA16(B1a) | PX448836 |
| 2021CV16-HZ-12 | Hangzhou | 2021 | Throat swab | CVA16(B1a) | PX448839 |
| 2021CV16-HZ-13 | Hangzhou | 2021 | Throat swab | CVA16(B1a) | PX448820 |
| 2021CV16-HZ-14 | Hangzhou | 2021 | Throat swab | CVA16(B1a) | PX448900 |
| 2021CV16-HZ-15 | Hangzhou | 2021 | Throat swab | CVA16(B1a) | PX448901 |
| 2021CV16-HZ-16 | Hangzhou | 2021 | Throat swab | CVA16(B1a) | PX448840 |
| 2021CV16-HZ-17 | Hangzhou | 2021 | Throat swab | CVA16(B1a) | PX448902 |
| 2021CV16-HZ-18 | Hangzhou | 2021 | Throat swab | CVA16(B1a) | PX448904 |
| 2021CV16-HZ-19 | Hangzhou | 2021 | Throat swab | CVA16(B1a) | PX448903 |
| 2021CV16-HZ-20 | Hangzhou | 2021 | Throat swab | CVA16(B1a) | PX448895 |
| 2021CV16-HZ-21 | Hangzhou | 2021 | Throat swab | CVA16(B1a) | PX448916 |
| 2021CV16-HZ-22 | Hangzhou | 2021 | Throat swab | CVA16(B1a) | PX448827 |
| 2021CV16-HZ-23 | Hangzhou | 2021 | Throat swab | CVA16(B1a) | PX448896 |
| 2021CV16-HZ-24 | Hangzhou | 2021 | Throat swab | CVA16(B1a) | PX448899 |
| 2021CV16-HZ-25 | Hangzhou | 2021 | Throat swab | CVA16(B1a) | PX448897 |
| 2021CV16-HZ-26 | Hangzhou | 2021 | Throat swab | CVA16(B1a) | PX448898 |
| 2021CV16-HZ-27 | Hangzhou | 2021 | Throat swab | CVA16(B1a) | PX448828 |
| 2021CV16-HZ-28 | Hangzhou | 2021 | Throat swab | CVA16(B1a) | PX448914 |
| 2021CV16-HZ-29 | Hangzhou | 2021 | Throat swab | CVA16(B1a) | PX448915 |
| 2021CV16-HZ-30 | Hangzhou | 2021 | Throat swab | CVA16(B1a) | PX448833 |
| 2021CV16-HZ-31 | Hangzhou | 2021 | Throat swab | CVA16(B1a) | PX448837 |
| 2021CV16-HZ-32 | Hangzhou | 2021 | Throat swab | CVA16(B1a) | PX448835 |
| 2021CV16-HZ-33 | Hangzhou | 2021 | Throat swab | CVA16(B1a) | PX448894 |
| 2021CV16-HZ-34 | Hangzhou | 2021 | Throat swab | CVA16(B1a) | PX448830 |
| 2021CV16-HZ-35 | Hangzhou | 2021 | Throat swab | CVA16(B1a) | PX448910 |
| 2021CV16-HZ-36 | Hangzhou | 2021 | Throat swab | CVA16(B1a) | PX448851 |
| 2021CV16-HZ-37 | Hangzhou | 2021 | Throat swab | CVA16(B1a) | PX448843 |
| 2021CV16-HZ-38 | Hangzhou | 2021 | Throat swab | CVA16(B1a) | PX448823 |
| 2021CV16-HZ-39 | Hangzhou | 2021 | Throat swab | CVA16(B1a) | PX448844 |
| 2021CV16-HZ-40 | Hangzhou | 2021 | Throat swab | CVA16(B1a) | PX448880 |
| 2021CV16-HZ-41 | Hangzhou | 2021 | Throat swab | CVA16(B1a) | PX448908 |
| **Sample name** | **Separate area** | **Sampling date** | **Specimen type** | **genotyping** | **GenBank accession** |
| 2021CV16-HZ-42 | Hangzhou | 2021 | Throat swab | CVA16(B1a) | PX448829 |
| 2021CV16-HZ-43 | Hangzhou | 2021 | Throat swab | CVA16(B1a) | PX448909 |
| 2022CV16-HZ-44 | Hangzhou | 2022 | Throat swab | CVA16(B1a) | PX448888 |
| 2022CV16-HZ-45 | Hangzhou | 2022 | Throat swab | CVA16(B1a) | PX448845 |
| 2022CV16-HZ-46 | Hangzhou | 2022 | Throat swab | CVA16(B1a) | PX448962 |
| 2022CV16-HZ-47 | Hangzhou | 2022 | Throat swab | CVA16(B1a) | PX448963 |
| 2022CV16-HZ-48 | Hangzhou | 2022 | Throat swab | CVA16(B1a) | PX448825 |
| 2022CV16-HZ-49 | Hangzhou | 2022 | Throat swab | CVA16(B1a) | PX448964 |
| 2022CV16-HZ-50 | Hangzhou | 2022 | Throat swab | CVA16(B1b) | PX448979 |
| 2022CV16-HZ-51 | Hangzhou | 2022 | Throat swab | CVA16(B1a) | PX448826 |
| 2022CV16-HZ-52 | Hangzhou | 2022 | Throat swab | CVA16(B1a) | PX448965 |
| 2022CV16-HZ-53 | Hangzhou | 2022 | Throat swab | CVA16(B1a) | PX448917 |
| 2022CV16-HZ-54 | Hangzhou | 2022 | Throat swab | CVA16(B1a) | PX448849 |
| 2022CV16-HZ-55 | Hangzhou | 2022 | Throat swab | CVA16(B1a) | PX448889 |
| 2022CV16-HZ-56 | Hangzhou | 2022 | Throat swab | CVA16(B1a) | PX448846 |
| 2022CV16-HZ-57 | Hangzhou | 2022 | Throat swab | CVA16(B1a) | PX448850 |
| 2022CV16-HZ-58 | Hangzhou | 2022 | Throat swab | CVA16(B1a) | PX448855 |
| 2022CV16-HZ-59 | Hangzhou | 2022 | Throat swab | CVA16(B1a) | PX448854 |
| 2022CV16-HZ-60 | Hangzhou | 2022 | Throat swab | CVA16(B1b) | PX448967 |
| 2022CV16-HZ-61 | Hangzhou | 2022 | Throat swab | CVA16(B1b) | PX448978 |
| 2022CV16-HZ-62 | Hangzhou | 2022 | Throat swab | CVA16(B1a) | PX448841 |
| 2022CV16-HZ-63 | Hangzhou | 2022 | Throat swab | CVA16(B1b) | PX448977 |
| 2022CV16-HZ-64 | Hangzhou | 2022 | Throat swab | CVA16(B1a) | PX448847 |
| 2022CV16-HZ-65 | Hangzhou | 2022 | Throat swab | CVA16(B1a) | PX448842 |
| 2022CV16-HZ-66 | Hangzhou | 2022 | Throat swab | CVA16(B1a) | PX448852 |
| 2022CV16-HZ-67 | Hangzhou | 2022 | Throat swab | CVA16(B1b) | PX448981 |
| 2022CV16-HZ-68 | Hangzhou | 2022 | Throat swab | CVA16(B1a) | PX448881 |
| 2022CV16-HZ-69 | Hangzhou | 2022 | Throat swab | CVA16(B1a) | PX448966 |
| 2022CV16-HZ-70 | Hangzhou | 2022 | Throat swab | CVA16(B1b) | PX448980 |
| 2022CV16-HZ-71 | Hangzhou | 2022 | Throat swab | CVA16(B1a) | PX448853 |
| 2022CV16-HZ-72 | Hangzhou | 2022 | Throat swab | CVA16(B1a) | PX448961 |
| 2022CV16-HZ-73 | Hangzhou | 2022 | Throat swab | CVA16(B1a) | PX448890 |
| 2022CV16-HZ-74 | Hangzhou | 2022 | Throat swab | CVA16(B1a) | PX448882 |
| 2022CV16-HZ-75 | Hangzhou | 2022 | Throat swab | CVA16(B1b) | PX448968 |
| 2022CV16-HZ-76 | Hangzhou | 2022 | Throat swab | CVA16(B1a) | PX448891 |
| 2022CV16-HZ-77 | Hangzhou | 2022 | Throat swab | CVA16(B1a) | PX448905 |
| 2022CV16-HZ-78 | Hangzhou | 2022 | Throat swab | CVA16(B1a) | PX448848 |
| 2022CV16-HZ-79 | Hangzhou | 2022 | Throat swab | CVA16(B1a) | PX448824 |
| 2022CV16-HZ-80 | Hangzhou | 2022 | Throat swab | CVA16(B1b) | PX448969 |
| 2022CV16-HZ-81 | Hangzhou | 2022 | Throat swab | CVA16(B1a) | PX448907 |
| 2022CV16-HZ-82 | Hangzhou | 2022 | Throat swab | CVA16(B1a) | PX448832 |
| 2022CV16-HZ-83 | Hangzhou | 2022 | Throat swab | CVA16(B1a) | PX448831 |
| **Sample name** | **Separate area** | **Sampling date** | **Specimen type** | **genotyping** | **GenBank accession** |
| 2023CV16-HZ-84 | Hangzhou | 2023 | Throat swab | CVA16(B1a) | PX448935 |
| 2023CV16-HZ-85 | Hangzhou | 2023 | Throat swab | CVA16(B1a) | PX448933 |
| 2023CV16-HZ-86 | Hangzhou | 2023 | Throat swab | CVA16(B1a) | PX448932 |
| 2023CV16-HZ-87 | Hangzhou | 2023 | Throat swab | CVA16(B1a) | PX448912 |
| 2023CV16-HZ-88 | Hangzhou | 2023 | Throat swab | CVA16(B1a) | PX448931 |
| 2023CV16-HZ-89 | Hangzhou | 2023 | Throat swab | CVA16(B1a) | PX448936 |
| 2023CV16-HZ-90 | Hangzhou | 2023 | Throat swab | CVA16(B1a) | PX448937 |
| 2023CV16-HZ-91 | Hangzhou | 2023 | Throat swab | CVA16(B1a) | PX448938 |
| 2023CV16-HZ-92 | Hangzhou | 2023 | Throat swab | CVA16(B1a) | PX448939 |
| 2023CV16-HZ-93 | Hangzhou | 2023 | Throat swab | CVA16(B1a) | PX448934 |
| 2023CV16-HZ-94 | Hangzhou | 2023 | Throat swab | CVA16(B1a) | PX448941 |
| 2023CV16-HZ-95 | Hangzhou | 2023 | Throat swab | CVA16(B1a) | PX448946 |
| 2023CV16-HZ-96 | Hangzhou | 2023 | Throat swab | CVA16(B1a) | PX448944 |
| 2023CV16-HZ-97 | Hangzhou | 2023 | Throat swab | CVA16(B1a) | PX448940 |
| 2023CV16-HZ-98 | Hangzhou | 2023 | Throat swab | CVA16(B1a) | PX448942 |
| 2023CV16-HZ-99 | Hangzhou | 2023 | Throat swab | CVA16(B1a) | PX448945 |
| 2023CV16-HZ-100 | Hangzhou | 2023 | Throat swab | CVA16(B1a) | PX448943 |
| 2023CV16-HZ-101 | Hangzhou | 2023 | Throat swab | CVA16(B1a) | PX448884 |
| 2023CV16-HZ-102 | Hangzhou | 2023 | Throat swab | CVA16(B1a) | PX448947 |
| 2023CV16-HZ-103 | Hangzhou | 2023 | Throat swab | CVA16(B1a) | PX448883 |
| 2023CV16-HZ-104 | Hangzhou | 2023 | Throat swab | CVA16(B1b) | PX448970 |
| 2023CV16-HZ-105 | Hangzhou | 2023 | Throat swab | CVA16(B1b) | PX448973 |
| 2023CV16-HZ-106 | Hangzhou | 2023 | Throat swab | CVA16(B1b) | PX448971 |
| 2023CV16-HZ-107 | Hangzhou | 2023 | Throat swab | CVA16(B1b) | PX448972 |
| 2023CV16-HZ-108 | Hangzhou | 2023 | Throat swab | CVA16(B1a) | PX448948 |
| 2023CV16-HZ-109 | Hangzhou | 2023 | Throat swab | CVA16(B1b) | PX448975 |
| 2023CV16-HZ-110 | Hangzhou | 2023 | Throat swab | CVA16(B1c) | PX448985 |
| 2023CV16-HZ-111 | Hangzhou | 2023 | Throat swab | CVA16(B1a) | PX448886 |
| 2023CV16-HZ-112 | Hangzhou | 2023 | Throat swab | CVA16(B1b) | PX448976 |
| 2024CV16-HZ-113 | Hangzhou | 2024 | Throat swab | CVA16(B1c) | PX448992 |
| 2024CV16-HZ-114 | Hangzhou | 2024 | Throat swab | CVA16(B1c) | PX449003 |
| 2024CV16-HZ-115 | Hangzhou | 2024 | Throat swab | CVA16(B1c) | PX449038 |
| 2024CV16-HZ-116 | Hangzhou | 2024 | Throat swab | CVA16(B1c) | PX449037 |
| 2024CV16-HZ-117 | Hangzhou | 2024 | Throat swab | CVA16(B1c) | PX448987 |
| 2024CV16-HZ-118 | Hangzhou | 2024 | Throat swab | CVA16(B1a) | PX448927 |
| 2024CV16-HZ-119 | Hangzhou | 2024 | Throat swab | CVA16(B1c) | PX449017 |
| 2024CV16-HZ-120 | Hangzhou | 2024 | Throat swab | CVA16(B1a) | PX448919 |
| 2024CV16-HZ-121 | Hangzhou | 2024 | Throat swab | CVA16(B1c) | PX448986 |
| 2024CV16-HZ-122 | Hangzhou | 2024 | Throat swab | CVA16(B1a) | PX448856 |
| 2024CV16-HZ-123 | Hangzhou | 2024 | Throat swab | CVA16(B1c) | PX448993 |
| 2024CV16-HZ-124 | Hangzhou | 2024 | Throat swab | CVA16(B1c) | PX448994 |
| 2024CV16-HZ-125 | Hangzhou | 2024 | Throat swab | CVA16(B1a) | PX448879 |
| **Sample name** | **Separate area** | **Sampling date** | **Specimen type** | **genotyping** | **GenBank accession** |
| 2024CV16-HZ-126 | Hangzhou | 2024 | Throat swab | CVA16(B1a) | PX448857 |
| 2024CV16-HZ-127 | Hangzhou | 2024 | Throat swab | CVA16(B1c) | PX449013 |
| 2024CV16-HZ-128 | Hangzhou | 2024 | Throat swab | CVA16(B1c) | PX449020 |
| 2024CV16-HZ-129 | Hangzhou | 2024 | Throat swab | CVA16(B1c) | PX449029 |
| 2024CV16-HZ-130 | Hangzhou | 2024 | Throat swab | CVA16(B1c) | PX448998 |
| 2024CV16-HZ-131 | Hangzhou | 2024 | Throat swab | CVA16(B1c) | PX448999 |
| 2024CV16-HZ-132 | Hangzhou | 2024 | Throat swab | CVA16(B1a) | PX448873 |
| 2024CV16-HZ-133 | Hangzhou | 2024 | Throat swab | CVA16(B1a) | PX448859 |
| 2024CV16-HZ-134 | Hangzhou | 2024 | Throat swab | CVA16(B1a) | PX448950 |
| 2024CV16-HZ-135 | Hangzhou | 2024 | Throat swab | CVA16(B1a) | PX448860 |
| 2024CV16-HZ-136 | Hangzhou | 2024 | Throat swab | CVA16(B1c) | PX448988 |
| 2024CV16-HZ-137 | Hangzhou | 2024 | Throat swab | CVA16(B1c) | PX449014 |
| 2024CV16-HZ-138 | Hangzhou | 2024 | Throat swab | CVA16(B1c) | PX449015 |
| 2024CV16-HZ-139 | Hangzhou | 2024 | Throat swab | CVA16(B1c) | PX449016 |
| 2024CV16-HZ-140 | Hangzhou | 2024 | Throat swab | CVA16(B1c) | PX449012 |
| 2024CV16-HZ-141 | Hangzhou | 2024 | Throat swab | CVA16(B1a) | PX448920 |
| 2024CV16-HZ-142 | Hangzhou | 2024 | Throat swab | CVA16(B1a) | PX448922 |
| 2024CV16-HZ-143 | Hangzhou | 2024 | Throat swab | CVA16(B1a) | PX448923 |
| 2024CV16-HZ-144 | Hangzhou | 2024 | Throat swab | CVA16(B1a) | PX448921 |
| 2024CV16-HZ-145 | Hangzhou | 2024 | Throat swab | CVA16(B1a) | PX448924 |
| 2024CV16-HZ-146 | Hangzhou | 2024 | Throat swab | CVA16(B1a) | PX448925 |
| 2024CV16-HZ-147 | Hangzhou | 2024 | Throat swab | CVA16(B1a) | PX448952 |
| 2024CV16-HZ-148 | Hangzhou | 2024 | Throat swab | CVA16(B1c) | PX449005 |
| 2024CV16-HZ-149 | Hangzhou | 2024 | Throat swab | CVA16(B1c) | PX449002 |
| 2024CV16-HZ-150 | Hangzhou | 2024 | Throat swab | CVA16(B1a) | PX448865 |
| 2024CV16-HZ-151 | Hangzhou | 2024 | Throat swab | CVA16(B1c) | PX449008 |
| 2024CV16-HZ-152 | Hangzhou | 2024 | Throat swab | CVA16(B1c) | PX449000 |
| 2024CV16-HZ-153 | Hangzhou | 2024 | Throat swab | CVA16(B1c) | PX449007 |
| 2024CV16-HZ-154 | Hangzhou | 2024 | Throat swab | CVA16(B1a) | PX448874 |
| 2024CV16-HZ-155 | Hangzhou | 2024 | Throat swab | CVA16(B1a) | PX448866 |
| 2024CV16-HZ-156 | Hangzhou | 2024 | Throat swab | CVA16(B1a) | PX448956 |
| 2024CV16-HZ-157 | Hangzhou | 2024 | Throat swab | CVA16(B1a) | PX448959 |
| 2024CV16-HZ-158 | Hangzhou | 2024 | Throat swab | CVA16(B1a) | PX448957 |
| 2024CV16-HZ-159 | Hangzhou | 2024 | Throat swab | CVA16(B1a) | PX448868 |
| 2024CV16-HZ-160 | Hangzhou | 2024 | Throat swab | CVA16(B1c) | PX449018 |
| 2024CV16-HZ-161 | Hangzhou | 2024 | Throat swab | CVA16(B1c) | PX449023 |
| 2024CV16-HZ-162 | Hangzhou | 2024 | Throat swab | CVA16(B1a) | PX448953 |
| 2024CV16-HZ-163 | Hangzhou | 2024 | Throat swab | CVA16(B1a) | PX448982 |
| 2024CV16-HZ-164 | Hangzhou | 2024 | Throat swab | CVA16(B1a) | PX448911 |
| 2024CV16-HZ-165 | Hangzhou | 2024 | Throat swab | CVA16(B1c) | PX448989 |
| 2024CV16-HZ-166 | Hangzhou | 2024 | Throat swab | CVA16(B1a) | PX448954 |
| 2024CV16-HZ-167 | Hangzhou | 2024 | Throat swab | CVA16(B1c) | PX448990 |
| **Sample name** | **Separate area** | **Sampling date** | **Specimen type** | **genotyping** | **GenBank accession** |
| 2024CV16-HZ-168 | Hangzhou | 2024 | Throat swab | CVA16(B1c) | PX448991 |
| 2024CV16-HZ-169 | Hangzhou | 2024 | Throat swab | CVA16(B1a) | PX448929 |
| 2024CV16-HZ-170 | Hangzhou | 2024 | Throat swab | CVA16(B1a) | PX448983 |
| 2024CV16-HZ-171 | Hangzhou | 2024 | Throat swab | CVA16(B1c) | PX449021 |
| 2024CV16-HZ-172 | Hangzhou | 2024 | Throat swab | CVA16(B1a) | PX448861 |
| 2024CV16-HZ-173 | Hangzhou | 2024 | Throat swab | CVA16(B1a) | PX448864 |
| 2024CV16-HZ-174 | Hangzhou | 2024 | Throat swab | CVA16(B1b) | PX448974 |
| 2024CV16-HZ-175 | Hangzhou | 2024 | Throat swab | CVA16(B1c) | PX448997 |
| 2024CV16-HZ-176 | Hangzhou | 2024 | Throat swab | CVA16(B1a) | PX448955 |
| 2024CV16-HZ-177 | Hangzhou | 2024 | Throat swab | CVA16(B1c) | PX448995 |
| 2024CV16-HZ-178 | Hangzhou | 2024 | Throat swab | CVA16(B1a) | PX448876 |
| 2024CV16-HZ-179 | Hangzhou | 2024 | Throat swab | CVA16(B1a) | PX448871 |
| 2024CV16-HZ-180 | Hangzhou | 2024 | Throat swab | CVA16(B1c) | PX449022 |
| 2024CV16-HZ-181 | Hangzhou | 2024 | Throat swab | CVA16(B1a) | PX448872 |
| 2024CV16-HZ-182 | Hangzhou | 2024 | Throat swab | CVA16(B1a) | PX448862 |
| 2024CV16-HZ-183 | Hangzhou | 2024 | Throat swab | CVA16(B1c) | PX449028 |
| 2024CV16-HZ-184 | Hangzhou | 2024 | Throat swab | CVA16(B1c) | PX449019 |
| 2024CV16-HZ-185 | Hangzhou | 2024 | Throat swab | CVA16(B1a) | PX448926 |
| 2024CV16-HZ-186 | Hangzhou | 2024 | Throat swab | CVA16(B1c) | PX449039 |
| 2024CV16-HZ-187 | Hangzhou | 2024 | Throat swab | CVA16(B1a) | PX448928 |
| 2024CV16-HZ-188 | Hangzhou | 2024 | Throat swab | CVA16(B1c) | PX449024 |
| 2024CV16-HZ-189 | Hangzhou | 2024 | Throat swab | CVA16(B1a) | PX448958 |
| 2024CV16-HZ-190 | Hangzhou | 2024 | Throat swab | CVA16(B1c) | PX449004 |
| 2024CV16-HZ-191 | Hangzhou | 2024 | Throat swab | CVA16(B1c) | PX449009 |
| 2024CV16-HZ-192 | Hangzhou | 2024 | Throat swab | CVA16(B1c) | PX449006 |
| 2024CV16-HZ-193 | Hangzhou | 2024 | Throat swab | CVA16(B1a) | PX448960 |
| 2024CV16-HZ-194 | Hangzhou | 2024 | Throat swab | CVA16(B1c) | PX449010 |
| 2024CV16-HZ-195 | Hangzhou | 2024 | Throat swab | CVA16(B1a) | PX448867 |
| 2024CV16-HZ-196 | Hangzhou | 2024 | Throat swab | CVA16(B1c) | PX449001 |
| 2024CV16-HZ-197 | Hangzhou | 2024 | Throat swab | CVA16(B1c) | PX449030 |
| 2024CV16-HZ-198 | Hangzhou | 2024 | Throat swab | CVA16(B1c) | PX449033 |
| 2024CV16-HZ-199 | Hangzhou | 2024 | Throat swab | CVA16(B1c) | PX449025 |
| 2024CV16-HZ-200 | Hangzhou | 2024 | Throat swab | CVA16(B1c) | PX449036 |
| 2024CV16-HZ-201 | Hangzhou | 2024 | Throat swab | CVA16(B1c) | PX449032 |
| 2024CV16-HZ-202 | Hangzhou | 2024 | Throat swab | CVA16(B1c) | PX449035 |
| 2024CV16-HZ-203 | Hangzhou | 2024 | Throat swab | CVA16(B1a) | PX448885 |
| 2024CV16-HZ-204 | Hangzhou | 2024 | Throat swab | CVA16(B1c) | PX449034 |
| 2024CV16-HZ-205 | Hangzhou | 2024 | Throat swab | CVA16(B1c) | PX449011 |
| 2024CV16-HZ-206 | Hangzhou | 2024 | Throat swab | CVA16(B1c) | PX449031 |
| 2024CV16-HZ-207 | Hangzhou | 2024 | Throat swab | CVA16(B1a) | PX448863 |
| 2024CV16-HZ-208 | Hangzhou | 2024 | Throat swab | CVA16(B1a) | PX448951 |
| 2024CV16-HZ-209 | Hangzhou | 2024 | Throat swab | CVA16(B1a) | PX448949 |
| **Sample name** | **Separate area** | **Sampling date** | **Specimen type** | **genotyping** | **GenBank accession** |
| 2024CV16-HZ-210 | Hangzhou | 2024 | Throat swab | CVA16(B1a) | PX448887 |
| 2024CV16-HZ-211 | Hangzhou | 2024 | Throat swab | CVA16(B1a) | PX448930 |
| 2024CV16-HZ-212 | Hangzhou | 2024 | Throat swab | CVA16(B1a) | PX448875 |
| 2024CV16-HZ-213 | Hangzhou | 2024 | Throat swab | CVA16(B1c) | PX449026 |
| 2024CV16-HZ-214 | Hangzhou | 2024 | Throat swab | CVA16(B1a) | PX448858 |
| 2024CV16-HZ-215 | Hangzhou | 2024 | Throat swab | CVA16(B1a) | PX448877 |
| 2024CV16-HZ-216 | Hangzhou | 2024 | Throat swab | CVA16(B1c) | PX449027 |
| 2024CV16-HZ-217 | Hangzhou | 2024 | Throat swab | CVA16(B1a) | PX448984 |
| 2024CV16-HZ-218 | Hangzhou | 2024 | Throat swab | CVA16(B1a) | PX448869 |
| 2024CV16-HZ-219 | Hangzhou | 2024 | Throat swab | CVA16(B1c) | PX448996 |
| 2024CV16-HZ-220 | Hangzhou | 2024 | Throat swab | CVA16(B1a) | PX448870 |
| 2024CV16-HZ-221 | Hangzhou | 2024 | Throat swab | CVA16(B1a) | PX448878 |

**Table S2 Information about the reference sequence of CV-A16 in this study**

| **GenBank accession** | **Strain name** | **Isolated country** | **Separation time** | **Genotype** |
| --- | --- | --- | --- | --- |
| U05876 | CAU05876-G10 | South Africa | 1951 | A |
| JQ746659 | CV-A16-genotypeA | Malaysia | 2012 | A |
| EU812514 | FY18 | China | 2008 | A |
| LT617106 | CVA16_C_CF172083_FRA_2012 | France | 2012 | B2 |
| LT617115 | CVA16_C_PAR190033_FRA_2014 | France | 2014 | B2 |
| LT617107 | CVA16_C_CF178025_FRA_2012 | France | 2012 | B2 |
| LT617105 | CVA16_C_CF350028_FRA_2011 | France | 2011 | B2 |
| LT617108 | CVA16_C_CF350028_FRA_2011 | France | 2011 | B2 |
| LT617113 | CVA16_C_LYO171046_FRA_2014 | France | 2014 | B2 |
| LT617114 | CVA16_C_PAR181046_FRA_2014 | France | 2014 | B2 |
| KC755231 | AH10-12 | China | 2010 | B1b |
| JN674176 | HN1662/HeN/CHN/2010 | China | 2010 | B1b |
| JN590244 | G20/YN/CHN/2010 | China | 2010 | B1b |
| GQ279371 | SZ/HK08-7/HK/CHN/2008 | China | 2008 | B1b |
| JX986741 | Wuhan0157/HuB/CHN/2011 | China | 2011 | B1b |
| HQ269389 | XM-CA16-3560/FJ/CHN/2009 | China | 2009 | B1b |
| KY425530 | R5/YN/CHN/2011 | China | 2011 | B1b |
| JX068830 | BJ11/03/BJ/CHN/2011 | China | 2011 | B1b |
| JX068827 | TS10/07/CHN/2010 | China | 2010 | B1b |
| JQ034149 | SH/CHN/2009/ CHN/2010 | China | 2010 | B1b |
| KU254598 | BJ14-4 | China | 2016 | B1b |
| KM215267 | CVA16/SZ29/CHN/2014 | China | 2014 | B1b |
| MH010199 | CVA16/Shenzhen469/CHN/2015 | China | 2015 | B1b |
| JQ746678 | PM-35210-06 | Malaysia | 2006 | B1c |
| JQ746672 | PM-1795457-07 | Malaysia | 2007 | B1c |
| LT617100 | CVA16_B_CF166109_FRA_2010 | France | 2010 | B1c |
| LT617103 | CVA16_B_CF312044_FRA_2010 | France | 2010 | B1c |
| LT617102 | CVA16_B_CF279014_FRA_2010 | France | 2010 | B1c |
| LT617104 | CVA16_B_CF223065_FRA_2011 | France | 2010 | B1c |
| KY792578 | CV-A16-A06-BLR-IN | India | 2013 | B1c |
| KY792576 | CV-A16-A01-BLR-IN | India | 2012 | B1c |
| KY792581 | CV-A16-A122-BLR-IN | India | 2015 | B1c |
| KY792579 | CV-A16-A10-BLR-IN | India | 2013 | B1c |
| KY792580 | CV-A16-A13-BLR-IN | India | 2017 | B1c |
| KY792583 | CV-A16-M02-BLR-IN | India | 2013 | B1c |
| KY792582 | CV-A16-A128-BLR-IN | India | 2015 | B1c |
| KY792577 | CV-A16-A02-BLR-IN | India | 2018 | B1c |
| MT212029 | XJ17-212/XJ/West/CHN/2017-10-04 | China | 2017 | B1c |
| MH780757 | oV18-026 | India | 2020 | B1c |
| JF738003 | THA-CA16-090 | Thailand | 2010 | B1a |
| **GenBank accession** | **Strain name** | **Isolated country** | **Separation time** | **Genotype** |
| JF738004 | THA-CA16-069 | Thailand | 2010 | B1a |
| JX068829 | TS10/08 | China | 2010 | B1a |
| KC755234 | XZ10-C-1 | China | 2010 | B1a |
| JQ354992 | Ningbo.CHN/028-2/2009 | China | 2009 | B1a |
| HQ423141 | KMM/08 | China | 2008 | B1a |
| KC755235 | ZJ10-48 | China | 2010 | B1a |
| GQ279368 | SZ/HK08-3 | China | 2008 | B1a |
| KF055242 | changchun075 | China | 2014 | B1a |
| JQ316639 | HQ09011181 | China | 2011 | B1a |
| KP289412 | CV-A16/P1014/2013/China | China | 2013 | B1a |
| JX068831 | BJ/11/11 | China | 2011 | B1a |
| KC755230 | AH10-2 | China | 2010 | B1a |
| KC755229 | ZJ10-73 | China | 2010 | B1a |
| LT617094 | CVA16_B_BUD25_HUN_2008 | France | 2008 | B1a |
| JQ746661 | PM-12284-99 | Malaysia | 1999 | B1a |
| JQ746660 | PM-00033-07 | Malaysia | 2007 | B1a |
| JQ746666 | PM-15765-00 | Malaysia | 2000 | B1a |
| MH010204 | CVA16/Shenzhen174/CHN/2017 | China | 2017 | B1a |
| JX839965 | Kor08-CVA16 | South Korea | 2008 | B1a |
| AF177911 | Tainan/5079/98 | China | 1998 | B1a |
