# Supplementary figures and images for: Epidemiology and genetic characteristics of coxsackievirus A16 associated with hand-foot-mouth disease in Hangzhou city, Zhejiang province from 2021 to 2024
Source: Front Microbiol. 2025 Nov 14;16:1698485. doi: 10.3389/fmicb.2025.1698485 (PMC12660211; doi:10.3389/fmicb.2025.1698485)

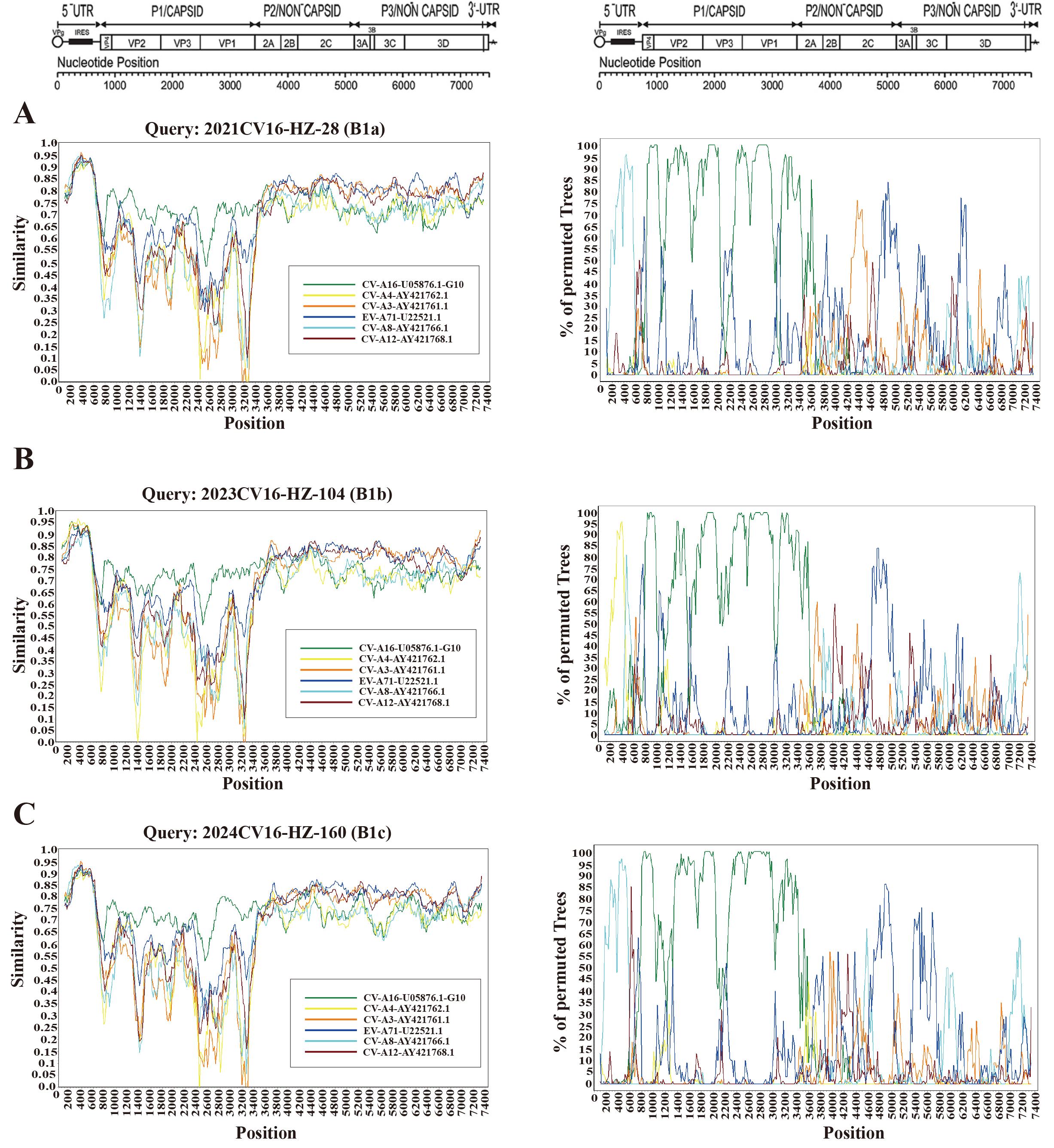

Supplement: Supplementary file 2 [file Image_1.jpeg]
